# Immune Responses to the Enduring Hypoxic Response Antigen Rv0188 Are Preferentially Detected in *Mycobacterium bovis* Infected Cattle with Low Pathology

**DOI:** 10.1371/journal.pone.0021371

**Published:** 2011-06-21

**Authors:** Gareth J. Jones, Chris Pirson, Hannah P. Gideon, Katalin A. Wilkinson, David R. Sherman, Robert J. Wilkinson, R. Glyn Hewinson, H. Martin Vordermeier

**Affiliations:** 1 Department of Bacteriology, Animal Health and Veterinary Laboratories Agency-Weybridge, Addlestone, Surrey, United Kingdom; 2 Institute of Infectious Diseases and Molecular Medicine, University of Cape Town, Cape Town, Western Cape, South Africa; 3 Mycobacterial Research, Medical Research Council National Institute for Medical Research, Mill Hill, London, United Kingdom; 4 Tuberculosis Program, Seattle Biomedical Research Institute, Seattle, Washington, United States of America; 5 Division of Medicine and Center for Molecular Microbiology and Infection, Imperial College, South Kensington, London, United Kingdom; University of Palermo, Italy

## Abstract

The DosR regulon and the Enduring Hypoxic Response (EHR) define a group of *M. tuberculosis* genes that are specifically induced in bacilli exposed *in vitro* to conditions thought to mimic the environment encountered by Mycobacteria during latent infection. Although well described in humans, latent mycobacterial infection in cattle remains poorly understood. Thus, the aim of this study was to identify antigens that may potentially disclose cattle with latent *M. bovis* infection. To this end, we initially screened 57 pools of overlapping peptides representing 4 DosR regulon and 29 EHR antigens for their ability to stimulate an immune response in whole blood from TB-reactor cattle using IFN-γ and IL-2 as readouts. All 4 DosR regulon proteins were poorly recognized (maximum responder frequency of 10%). For the EHR antigens, both IFN-γ and IL-2 revealed similar response hierarchies, with responder frequencies ranging from 54% down to 3% depending on the given EHR antigen. Furthermore, these results demonstrated that responses in the infected cattle were largely IFN-γ biased. To support the concept for their role in latency, we evaluated if EHR antigen responses were associated with lower pathology. The EHR antigen Rv0188 was recognised predominantly in animals presenting with low pathology scores, whereas responses to ESAT-6/CFP-10 or the other EHR antigens tested were prevalent across the pathology spectrum. However, when we determined the production of additional cytokines induced by the *M. bovis* antigens PPD-B or ESAT-6/CFP-10, we detected significantly greater PPD-B-induced production of the pro-inflammatory cytokine IL-1β in animals recognizing Rv0188 (i.e. those with limited or no pathology). Thus, these results are consistent with the idea that responses to Rv0188 may identify a subset of animals at early stages of infection or in which disease progression may be limited.

## Introduction

Tuberculosis (TB) is a mycobacterial infection that affects numerous species of mammals and poses a major global threat to both public health and the farming industry. Following exposure to *Mycobacterium tuberculosis* (*M. tb*), only a small percentage of individuals (5 to 10%) will progress to a clinically active stage of disease within the first two years, with symptoms including fever, weight loss, coughing, the presence of acid-fast bacilli in sputa or other clinical samples and abnormal chest radiographs. In contrast, the majority of *M. tb*-infected individuals are able to control disease progression through the induction of a robust cell-mediated immune response. However, despite the absence of clinical disease, mycobacteria remain viable in these individuals for many years [1]. More importantly, these “latent” mycobacterial infections have the potential to reactivate to active disease [2], probably as a consequence of events resulting in host immunosupression [2], [3], [4]. Based on the tuberculin skin test, it is thought that one-third of the world's population is latently infected with *M. tb*, which presents an enormous reservoir from which contagious TB disease may arise.

Latent mycobacterial infection constitutes a complex interaction between pathogen and host resulting in limited bacterial growth. Studies in mice have demonstrated that inhibition of bacterial growth in the lungs is preceded by increased expression of key host-immunity genes, such as IFN-γ and inducible nitric oxide (NO) synthase-2 [5]. In vitro culture of tubercle bacilli in IFN-γ-activated macrophages, or in the presence of low-dose NO and hypoxia (conditions thought to mimic those encountered by mycobacteria during the latent stage of infection) results in expression of a group of 48 genes known as the dormancy (DosR) regulon [6], [7]. More recently, studies in *M. tb*-infected individuals have revealed that the gene products of several DosR regulon genes are immunogenic, inducing T-cell proliferation and/or IFN-γ production [8], [9], [10], [11]. Furthermore, immune responses to the DosR antigen Rv2031c have been shown to be largely restricted to latently infected individuals [12], [13]. However, as other studies have shown conflicting results [14], [15], the possible use of the Rv2031c antigen as a diagnostic agent to differentiate between active and latent disease remains unclear. In addition to the DosR regulon, growth of mycobacteria in hypoxic conditions also induces at later time points (4–7 days) the expression of a further set of approximately 200 genes, known as the Enduring Hypoxic Response (EHR) [16]. Given that several EHR antigens induce IFN-γ and/or IL-2 production in PBMC from *M. tb*-infected individuals [14], the EHR may constitute an alternative set of stage-specific antigens with possible potential for differential diagnosis in TB infection.

Bovine tuberculosis (BTB) caused by the bacterial pathogen *Mycobacterium bovis* (*M. bovis*) continues to pose a major economic and animal health problem for the farming community, and the zoonotic potential of BTB still remains a concern in countries with little or no control policies. Unlike human *M. tb* infection, it remains unclear whether different stages of active versus latent infection also occur in *M. bovis*-infected cattle (reviewed in [17], [18], [19]). However, the existence of: (i) IFN-γ test positive animals that lack tuberculous lesions [20]; (ii) animals that culture positive in the absence of lesions [21]; or (iii) IFN-γ test positive but skin test negative animals that later convert to skin test positive and present with lesions (reviewed in [18], at least support the concept of BTB latency and subsequent reactivation in cattle. Indeed, studies have demonstrated reactivation of latent tuberculosis in humans infected with *M. bovis* [22]. Thus, if latent BTB does exist in a similar manner to that in humans, it would pose a serious complication to the eradication of the disease in cattle.

As mentioned, there is a large interest in developing diagnostic tests capable of differentiating active versus latent mycobacterial infections. Although the immunogenicity of stage-specific antigens has been investigated in human TB patients, no such evaluation has to date been performed in BTB cases. Thus, the aim of this present study was to investigate the immunogenicity of the DosR and EHR antigens using a cattle model of *M. bovis* infection. Our results identified several immunogenic EHR antigens, with responses to one such antigen (Rv0188) associated with a subset of animals at early stages of infection or in which disease progression may be limited.

## Methods

### Ethics Statement

All procedures were conducted within the limits of the Animal (Scientific Procedures) Act 1986, and approved by the United Kingdom Home Office and by the Veterinary Laboratories Agency (VLA) ethical review committee (Home Office Licence number 70/6775).

### Cattle

All animals were housed at the VLA at the time of blood sampling. In total, heparinised blood samples were obtained from 38 naturally infected, single intradermal comparative cervical tuberculin (SICCT) test-positive reactors from herds known to have bovine tuberculosis (BTB) as determined by government veterinarians of the Animal Health Agency. All TB-reactor animals were subjected to a detailed post-mortem examination and the severity of pathology was scored according to a semi-quantitative system as previously described [23].

### Production and preparation of peptides and antigens

Four proteins of the DosR regulon and 29 proteins of the Enduring Hypoxic Response (Table S1) were selected for antigen screening experiments. The DosR regulon antigens were selected based on their immunogenicity in human studies [8]. The selection of EHR antigens was based on a bioinformatic analysis (Gideon et al. in preparation). This included consideration of the fold induction and relative abundance of transcripts at 96 and 168 hours in hypoxic compared to oxygenated axenic cultures, number and affinity of MHC Class II binding peptides per molecule, genomic organisation and predicted function. Peptides representing the selected antigens were commercially produced (Mimotopes Pty Ltd, Clayton, Australia) as 20-mers overlapping by 12 amino acids, dissolved in RPMI 1640, formulated into peptide pools and used at a final concentration of 2–10 µg/ml/peptide. In total, 57 peptide pools containing a total of 855 peptides were evaluated. Peptides from ESAT-6 and CFP-10 were formulated to obtain a peptide cocktail as previously described [24] and were used at a final concentration of 5 µg/ml/peptide. Bovine tuberculin (PPD-B) was supplied by the Tuberculin Production Unit at the Veterinary Laboratories Agency, Weybridge, Surrey, UK and was used at a final concentration of 10 µg/ml. Staphylococcal enterotoxin B (SEB; Sigma-Aldrich, UK) was included as a positive control at a final concentration of 1 µg/ml, while RPMI 1640 alone served as a negative control.

### Stimulation of whole blood cultures

Whole blood aliquots (250 µl) were added in duplicate to antigen in 96-well plates and incubated at 37°C in the presence of 5% CO_2_ for 24 hours, following which plasma supernatants were harvested and stored at −80°C until required. The EHR antigens were screened in two batches, using either 36 or 19 TB reactor animals.

### Measurement of cytokine levels in plasma-supernatant

Quantification of IFN-γ in the plasma supernatant was determined using the Bovigam ELISA kit (Prionics AG, Switzerland) according to the manufacturer's instructions. A result was considered positive if the optical density at 450 nm (OD_450_) with antigen minus the OD_450_ without antigen (ΔOD_450_) was ≥0.1 in both of the duplicate wells. IL-2 production was determined by the ability to sustain proliferation of Concanavalin A (ConA)-induced lymphoblasts as previously described [25]. A result was considered positive if the stimulation index (SI; proliferation with antigen divided by proliferation without stimulus) was greater than 3. Lastly, simultaneous detection of IFN-γ, IL-1β, IL-4, IL-10, IL-12, MIP-1β and TNF-α was performed using a cytokine multiplex assay (Meso-Scale Discovery (MSD), Gaithersburg, MD, USA) as previously described [26].

### Statistical analysis

Statistical analysis was performed using GraphPad Instat 3 software (GraphPad Software Inc., USA).

## Results

**Figure 1:**
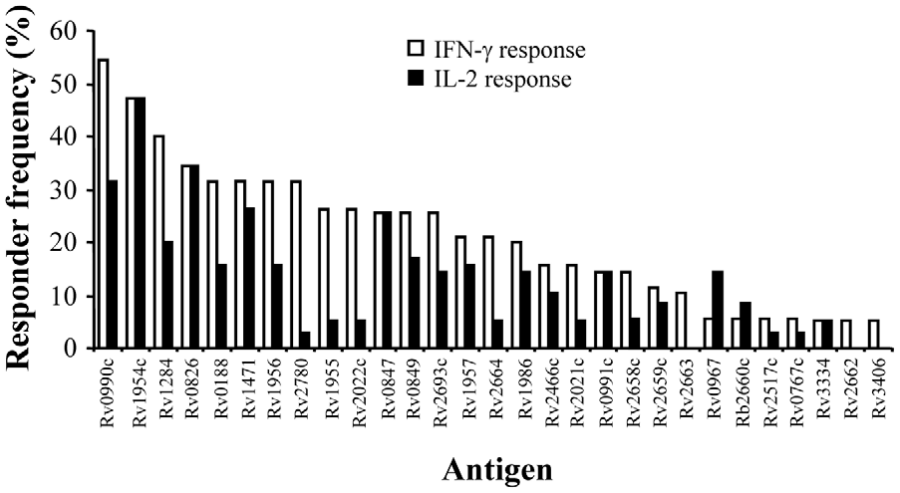
IL-2 and IFN-γ response hierarchy after stimulation with EHR antigens. Responses were established using blood samples from at least 19 TB-reactor animals. IFN-γ responses were determined using the BOVIGAM kit, IL-2 responses using a bio-assay (see Materials and Methods). Results are shown as responder frequencies (proportion of animals tested that gave a positive response). For comparison, the responder frequencies for ESAT-6/CFP-10 using IFN-γ and IL-2 as a readout were 89% and 84% respectively.

To investigate the immunogenicity of potential stage-specific antigens, a total of 57 pools of overlapping peptides representing 4 DosR regulon and 29 EHR antigens (Table S1) were screened for their ability to stimulate an immune response in whole blood from TB-reactor cattle. As initial experiments revealed that the DosR regulon proteins were poorly recognized (maximum responder frequency of 10%, Table S1), we focused our attention on the panel of EHR antigens (Figure 1). All 29 EHR antigens induced an IFN-γ response in at least one animal, with overall responder frequencies ranging from 5% up to 54% depending on the antigen. By contrast, three EHR antigens failed to induce an IL-2 response in any of the animals studied, while the remaining antigens were recognized to a variable degree with responder frequencies ranging from 3% up to 47%. In general, the hierarchy of response to the EHR antigens were similar irrespective of the cytokine readout. However, noticeable exceptions to this were Rv2780, Rv1955 and Rv2022c, which were all placed in the top ten antigens using IFN-γ as a readout but dropped to lower rankings when IL-2 was used as a readout (Figure 1 and Table S2). Furthermore, responses in TB-reactor animals were largely IFN-γ biased, as evidenced by higher responder frequencies to the majority of EHR antigens when using a IFN-γ as a readout compared to IL-2. Having demonstrated a hierarchy in responses to the panel of EHR antigens, we selected the top 5 most frequently recognised antigens (Rv0990c, Rv1954c, Rv1284, Rv0826 and Rv0188) for further analysis.

**Figure 2:**
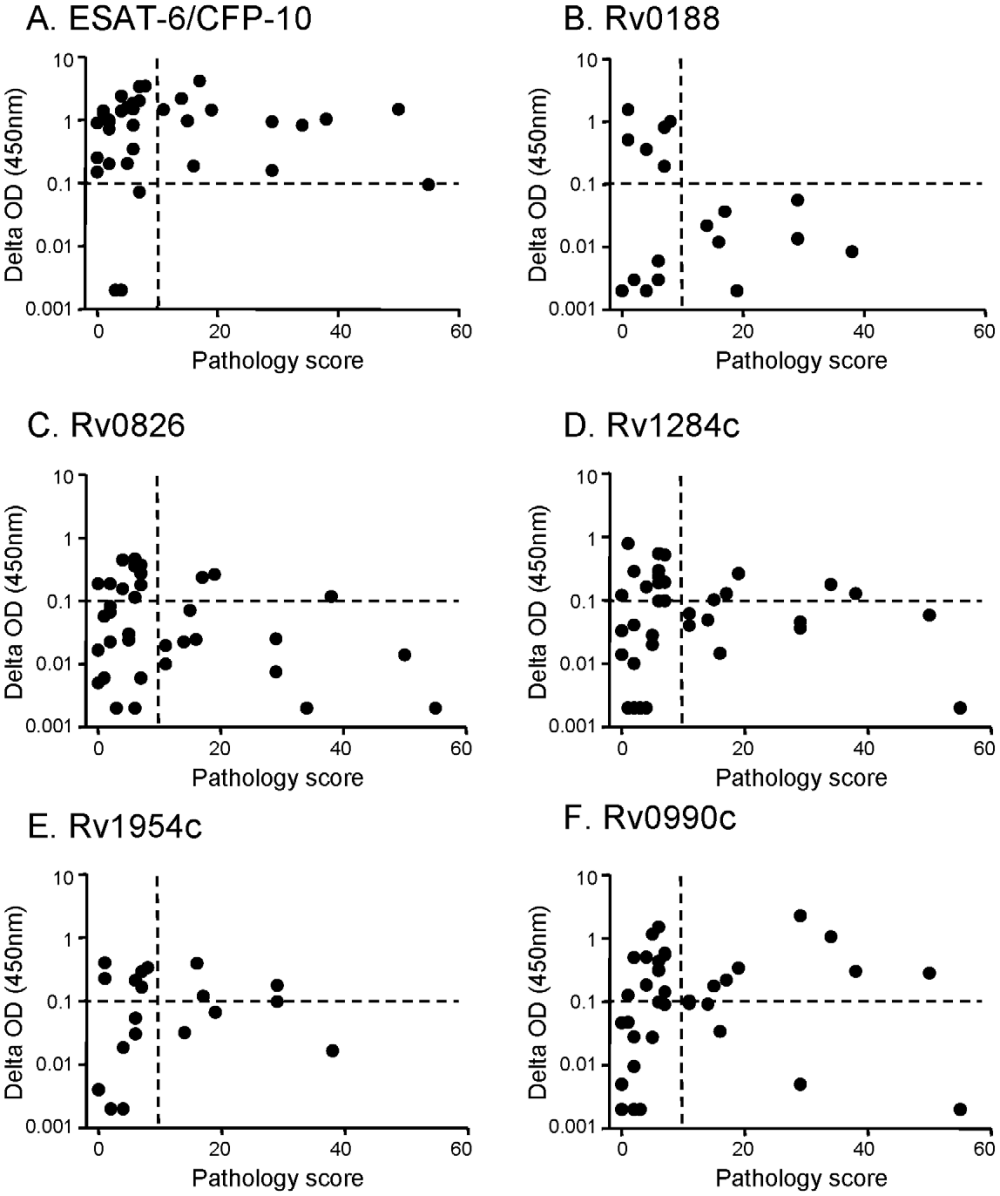
Comparison of antigen specific IFN-γ responses with pathology scores. Antigen induced IFN-γ responses were plotted in relation to the pathology score, with each symbol representing a single animal. Horizontal and vertical dotted lines define the cut offs for IFN-γ responders/non-responders and low pathology/high pathology respectively. Responses to ESAT-6/CFP-10 (A), Rv0188 and Rv1954c (B and E) and Rv0826, Rv1284c and Rv0990c (C, D and F) were evaluated in 38, 19 and 36 animals respectively.

**Figure 3:**
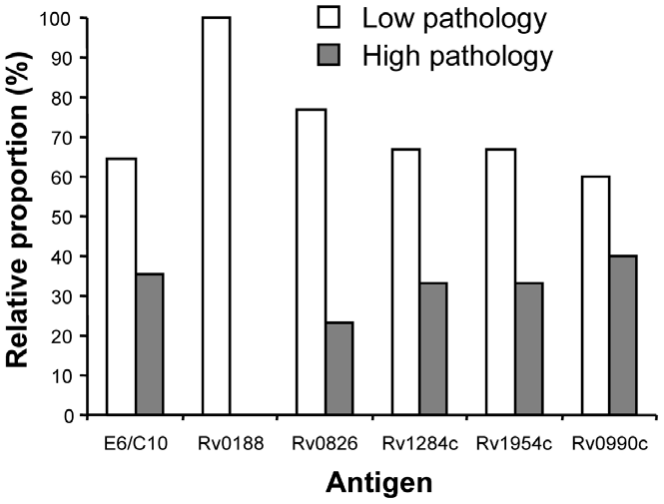
IFN-γ responses to Rv0188 were observed only in animals with low pathology scores. Graph represents the relative proportion of animals responding to either ESAT-6/CFP-10 (n = 34), Rv0188 (n = 6), Rv0826 (n = 13), Rv1284 (n = 15), Rv1954c (n = 9) or Rv0990c (n = 20) that showed either low or high pathology.

Although well described in humans, latent mycobacterial infection in cattle remains poorly classified. In order to define cattle with possible latent infection, a detailed post mortem examination was performed on all animals to assess the level of active disease. The presence of visible lesions in the lymph nodes of the upper and lower respiratory tract, lung tissue, mesenteric lymph nodes and all major body organs were recorded and the severity of pathology was scored for each animal according to a semi-quantitative system as previously described [23]. A spectrum of pathology was noted, with scores ranging from 0 (i.e. no visible lesions) to 55 (multiple lesions). For the purpose of this study, the animals were classified as having either low pathology (i.e. scores of less than 10) or high pathology (i.e. scores greater than 10). Comparison of pathology scores with immune responses to the *M. bovis* antigen ESAT-6/CFP-10 cocktail (Figure 2A) revealed positive IFN-γ responses in animals exhibiting either low (upper left quadrant) or high (upper right quadrant) pathology. However, positive IFN-γ responses to the EHR antigen Rv0188 were only evident in animals exhibiting low pathology (Figure 2B). Indeed, of the 12 animals with low pathology scores, six (50%) responded to Rv0188. In contrast to Rv0188, positive immune responses to the other four selected EHR antigens (Figure 2C–F) were seen across the pathology spectrum. Of the animals that responded to ESAT-6/CFP-10, approximately two-thirds exhibited low pathology while one-third exhibited high pathology (Figure 3). Similar frequency distributions were also evident in animals responding to the EHR antigens Rv0826, Rv1284c, Rv1954c or Rv0990c. Uniquely, animals responding to Rv0188 were comprised solely of those with low pathology.

**Figure 4:**
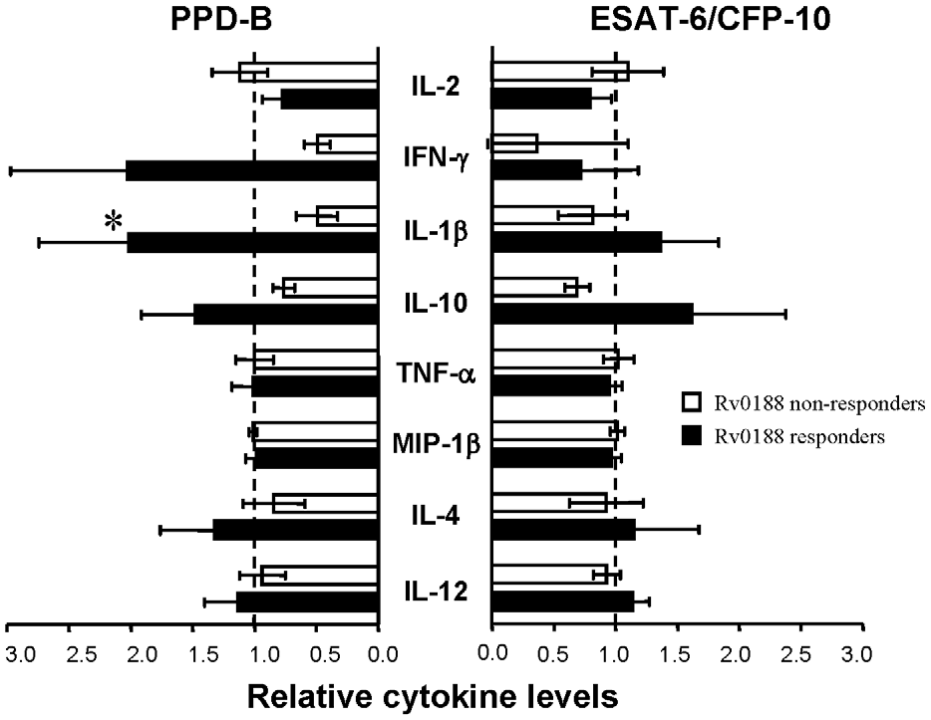
Elevated cytokine production in Rv0188-responder animals. Graph shows the relative cytokine levels (mean ± SEM) in Rv0188 responder animals (n = 6, filled bars) and Rv0188 non-responder animals (n = 12, open bars). Data are normalized to the mean cytokine concentration for all animals. Mean PPD-B induced cytokine concentrations were: 2.56 ng/ml (IL-1β); 0.52 ng/ml (IL-4); 18.04 units/ml (IL-10); 36.85 units/ml (IL-12); 64.47 ng/ml (MIP-1β); 15.67 ng/ml (IFN-γ); 3.99 ng/ml (TNF-α); and 26.76 SI (IL-2). Mean ESAT-6/CFP-10 induced cytokine concentrations were: 0.65 ng/ml (IL-1β); 0.43 ng/ml (IL-4); 9.44 units/ml (IL-10); 17.28 units/ml (IL-12); 53.77 ng/ml (MIP-1β); 6.90 ng/ml (IFN-γ); 2.73 ng/ml (TNF-α); and 11.61 SI (IL-2). * p<0.05 on non-normalised raw data, Unpaired T Test.

Having shown that positive responses to the EHR antigen Rv0188 identified cattle with only low pathology scores, we next investigated the immune profile of these animals in response to stimulation with other *M. bovis* antigens (PPD-B or ESAT-6/CFP-10). The antigen-induced cytokine concentrations for the individual animals are shown in Figure S1, while the relative cytokine levels for each group (Rv0188-responders and Rv0188 non-responders) are depicted in Figure 4. For the majority of cytokines induced by either PPD-B or ESAT-6/CFP-10, similar relative levels were seen between the two groups. However, there was a trend for greater IL-10, IL-1β and IFN-γ production in Rv0188 responder animals, which was most evident for PPD-B-driven responses, where greater IL-1β production in Rv0188 responder animals achieved statistical significance.

## Discussion

The development of a test capable of differentiating active and latent mycobacterial infections would have major implications to the management and control of tuberculosis in both public health and the farming industry. Such tests would likely involve the detection of immune responses to stage specific antigens, and several potential candidates have been identified for human-*M. tb* infections (including the DosR regulon antigens Rv1733c, Rv2029c, Rv2627c and Rv2628) based on their preferential detection in skin test positive individuals that lack clinical signs of active TB [8], [10], [11]. Thus, to evaluate if these antigens would be beneficial in the setting of BTB, we investigated the immunogenicity of Rv1733c, Rv2029c, Rv2627c and Rv2628 using whole blood cultures from skin test positive, *M. bovis* infected cattle. Disappointingly, these antigens were recognised, if at all, by a substantially lower proportion of skin test positive cattle compared to skin test positive humans [8]. Given that there is at least a 98% identity in amino acid sequence for these proteins between *M. tb* and *M. bovis* (strain AF2122/97), it is unlikely that the absence of T-cell responses in cattle is due to epitope variation between the two mycobacterial species. Furthermore, RNA transcript levels for all four DosR regulon genes were similar between *M. tb* (strain H37Rv) and *M. bovis* (BCG) grown *in vitro* in hypoxic conditions [9]. However, as BCG represents an attenuated form of *M. bovis*, it is unknown if wild type *M. bovis* strains would exhibit similar gene profiles in hypoxic conditions. The immunogenicity responses presented herein are consistent with previous studies demonstrating lack of responses to DosR regulon antigens in *M. bovis* BCG vaccinated mice and humans [9], [27]. However, again it remains unclear in these studies if this lack of response is due to *M. bovis* mycobacterium *per se* (as apposed to *M. tb*), or as a consequence of vaccination with an attenuated strain.

In contrast to the DosR regulon antigens, our results demonstrated that *M. bovis*-infected cattle more frequently recognized several EHR antigens. The immunogenicity of three EHR antigens (Rv1986, Rv2658c and Rv2659c) has also been recently described in *M. tb*-infected humans [14]. In this study, the proportion of individuals recognizing either Rv1986 or Rv2659c appeared to be greater when IL-2 was used as a readout for T-cell activation compared to IFN-γ. Indeed, almost all individuals studied mounted an IL-2 response to Rv1986. Furthermore, the majority of CD4+ T cells recognizing Rv1986 produced only IL-2, expressed CD27 but lacked CD45RA, leading to the possibility that this EHR antigen is the target of long lived central memory CD4+ T-cells. In contrast, our results demonstrated that: (i) Rv1986 induced IL-2 production in a considerably lower proportion (14%) of *M. bovis*-infected cattle; and (ii) immune responses to the remaining EHR antigens were largely IFN-γ biased. Thus, we speculate that in our TB-reactor cattle, the majority of EHR antigens studied are targeting effector CD4+ T cells, although we acknowledge that further flow cytometric data would be required to support this concept.

Although initially identified during *in vitro* studies culturing *M. tb* in conditions thought to mimic those encountered during latent mycobacterial infection (e.g. the presence of NO and hypoxia), it is unlikely that gene products of the DosR regulon and the EHR are truly restricted to a persistent, non-replicating stage of mycobacterial infection [28]. In support, immune responses to some DosR regulon and EHR antigens have been demonstrated in *M. tb*-infected individuals during both latent and active stages of disease [8], [14], [29], [30]. Our data from *M. bovis*-infected cattle would also support this concept, given that animals recognizing the majority of the immunogenic EHR antigens were not restricted to the low pathology group (Figure 3).

In human infections, latent TB is defined as evidence of immunological priming to mycobacterial antigens in the absence of clinical signs of active disease. Recently, it has been proposed that in contrast to a bimodal classification of distinct latent and active stages, TB infection should be viewed as a continuous spectrum where designations of latent and active disease correspond to partially overlapping responses to infection [31]. In this context, low pathology is either indicative of early infection or a robust host response (possibly correlating with latency). In the setting of *M. bovis* and cattle, latent mycobacterial infection remains poorly classified, although it has been suggested that skin or IFN-γ test positive animals that present with no visible lesions (NVL) may represent latently infected cattle [17]. Unfortunately in our study, only a small number of animals (3 out of 38) presented with no visible lesions, making it difficult to make meaningful comparisons between potentially latent and non-latent animals. Thus, for the purpose of this study, the animals were classified as having either low or high pathology. Using this classification, we demonstrated that Rv0188-specific IFN-γ responses were restricted to the low pathology group, leading us to hypothesize that this antigen may be an immune target during “latent” BTB. To explore this, we investigated the immune profile of Rv0188-responsive animals following stimulation with other *M. bovis* antigens (PPD-B or ESAT-6/CFP-10), reasoning that potentially latent infected animals would exhibit lower levels of cytokine production (and in particular pro-inflammatory cytokines such as MIP-1β, TNF-α and IL-1β) than those with active disease. This reasoning is supported in part by the results of previous studies in both humans and cattle, demonstrating that greater ESAT-6 and/or CFP-10-induced immune responses were associated with the severity of disease pathology [23], [29]. However, our results demonstrated that for the majority of cytokines, ESAT-6/CFP-10 induced similar levels of production between the Rv0188 responsive and Rv0188 non-responsive groups.

The results of our study may be interpreted in several ways. Firstly, given that responses to Rv0188 were only seen in animals with low pathology, it is possible that immune responses targeted directly to this antigen may contribute to the prevention of disease progression. Alternatively, responses to Rv0188 may be a biological marker identifying animals which, by targeting other mycobacterial proteins, are capable of limiting *M. bovis* infection. In support, our results show greater levels of PPD-B-induced IFN-γ and IL-1β production in the Rv0188 responsive animals. Lastly, it is possible that the animals exhibiting low pathology may represent cases of recent infection, in which disease progression is at an early phase. In this scenario, responses to Rv0188 may identify a subset of animals at early stages of infection. However, given that the animals studied were natural field reactors, it is unclear precisely how long these animals have been infected, and more controlled studies using experimentally infected animals are required to confirm any possible association between Rv0188 responses and early infection. Whatever the scenario, it most be noted that only half of the animals in the low pathology group were responsive to Rv0188, which highlights a limitation in using responses to this antigen alone in detecting animals with limited disease progression and/or at early stages of infection.

As previously mentioned, it has been suggested that the detection of primed cellular immunity to the mycobacterium in the absence of visible lesions (i.e. NVL) may disclose cattle with latent *M. bovis* infection [17]. The current selection criteria for inclusion of *M. bovis*-infected animals in our studies is a positive skin test reaction. However, as evidenced in this study, very few animals selected in this manner were NVL. It has been shown that IFN-γ positive and/or skin test negative animals can later convert to skin test positive and present lesions at slaughter (reviewed in [18]), leading to the speculation that these animals may have been latently infected when first identified but subsequently underwent reactivation of the disease. Thus, in future studies, we aim to target skin test negative but IFN-γ positive animals in a hope to identify a larger proportion of NVL animals required for evaluation of stage-specific antigens in BTB.

## Supporting Information

Table S1 Details of the DosR regulon and EHR proteins studied.
(XLS)

Table S2 Recognition of the EHR antigens using different cytokines readouts.
(DOC)

Figure S1 ***M. bovis*** **antigen-stimulated cytokine production in Rv0188-responder and non-responder animals.** Graph shows the levels of PPD-B and ESAT-6/CFP-10-stimulated cytokine production in Rv0188-responder (closed circles) and Rv0188-non-responder (open circles) animals. Each symbol represents a single animal. * p<0.05, Unpaired T Test.
(TIF)
