# Supplementary material for: Immune Responses to the Enduring Hypoxic Response Antigen Rv0188 Are Preferentially Detected in Mycobacterium bovis Infected Cattle with Low Pathology
Source: PLoS One. 2011 Jun 21;6(6):e21371. doi: 10.1371/journal.pone.0021371 (PMC3119702; doi:10.1371/journal.pone.0021371)
Supplement: Table S2 — Recognition of the EHR antigens using different cytokines readouts. (DOC) [file pone.0021371.s002.doc]

**Table S2. Recognition of the EHR antigens using different cytokines readouts.**

| Antigen | IFN-γ readout | | IL-2 readout | |
| --- | --- | --- | --- | --- |
|  | Frequency (%) | Rank | Frequency (%) | Rank |
| Rv0990c | 54 | 1 | 31 | 3 |
| Rv1954c | 47 | 2 | 47 | 1 |
| Rv1284 | 40 | 3 | 20 | 6 |
| Rv0826 | 34 | 4 | 34 | 2 |
| Rv0188 | 32 | 5 | 16 | 8 |
| Rv1471 | 32 | 5 | 26 | 4 |
| Rv1956 | 32 | 5 | 16 | 8 |
| Rv2780 | 31 | 8 | 3 | 24 |
| Rv1955 | 26 | 9 | 5 | 19 |
| Rv2022c | 26 | 9 | 5 | 19 |
| Rv0847 | 26 | 9 | 26 | 4 |
| Rv0849 | 26 | 9 | 17 | 7 |
| Rv2693c | 26 | 9 | 14 | 11 |
| Rv1957 | 21 | 14 | 16 | 8 |
| Rv2664 | 21 | 14 | 5 | 19 |
| Rv1986 | 20 | 16 | 14 | 11 |
| Rv2466c | 16 | 17 | 11 | 15 |
| Rv2021c | 16 | 17 | 5 | 19 |
| Rv0991c | 14 | 19 | 14 | 11 |
| Rv2658c | 14 | 19 | 6 | 18 |
| Rv2659c | 11 | 21 | 9 | 16 |
| Rv2663 | 11 | 21 | 0 | 27 |
| Rv0967 | 6 | 23 | 14 | 11 |
| Rv2660c | 6 | 23 | 9 | 16 |
| Rv2517c | 6 | 23 | 3 | 24 |
| Rv0767c | 6 | 23 | 3 | 24 |
| Rv3334 | 5 | 27 | 5 | 19 |
| Rv2662 | 5 | 27 | 0 | 27 |
| Rv3406 | 5 | 27 | 0 | 27 |
