# Supplementary figures and images for: Immune Responses to the Enduring Hypoxic Response Antigen Rv0188 Are Preferentially Detected in Mycobacterium bovis Infected Cattle with Low Pathology
Source: PLoS One. 2011 Jun 21;6(6):e21371. doi: 10.1371/journal.pone.0021371 (PMC3119702; doi:10.1371/journal.pone.0021371)

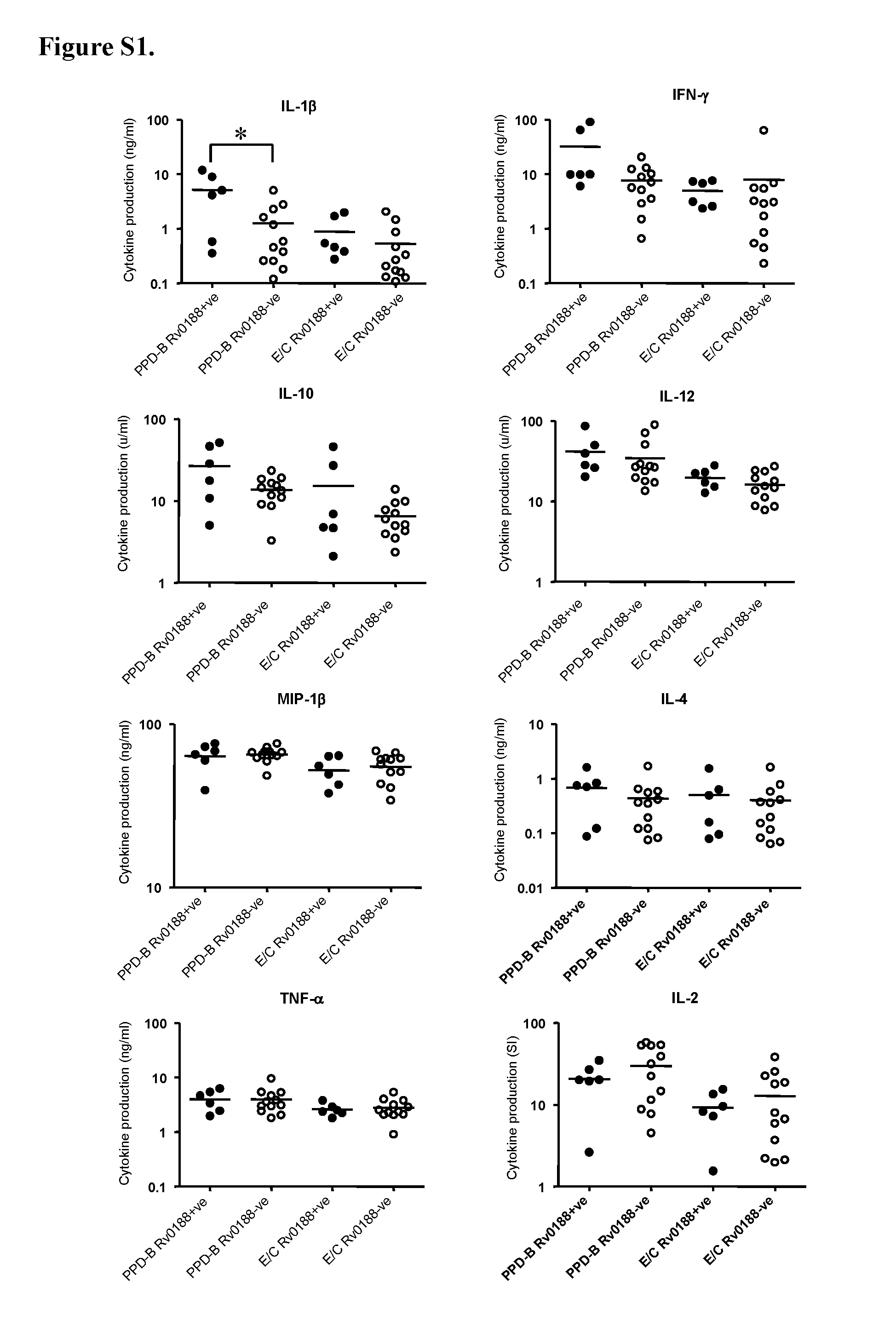

Supplement: Figure S1 — M. bovis antigen-stimulated cytokine production in Rv0188-responder and non-responder animals. Graph shows the levels of PPD-B and ESAT-6/CFP-10-stimulated cytokine production in Rv0188-responder (closed circles) and Rv0188-non-responder (open circles) animals. Each symbol represents a single animal. * p<0.05, Unpaired T Test. (TIF) [file pone.0021371.s003.tif]
